# 

**Published:** 1904-08

**Authors:** 


### Society Notes.

The Thirteenth Annual Session of the Mississippi Valley Medical Association will be held at Cincinnati, Ohio, October 11, 12, 13, 1904, under the presidency of Dr. Hugh T. Patrick of Chicago. The headquarters and meeting places will be at the Grand Hotel. The annual orations will be delivered by Dr. Wm. J. Mayo of Rochester, Minn., in Surgery, and Dr. C. Travis Drennen of Hot Springs, Ark., in Medicine. Request for places upon the program, or information in regard to the meeting, can be had by addressing the Secretary, Dr. Henry Enos Tuley, Louisville, Ky., or the Assistant Secretary, Dr. S. C. Stanton, Masonic Temple, Chicago, Ill. The usual railroad rates will be in effect.

